# The Role of One-Carbon Metabolism and Methyl Donors in Medically Assisted Reproduction: A Narrative Review of the Literature

**DOI:** 10.3390/ijms25094977

**Published:** 2024-05-02

**Authors:** Konstantinos Sfakianoudis, Athanasios Zikopoulos, Sokratis Grigoriadis, Nikolaos Seretis, Evangelos Maziotis, George Anifandis, Paraskevi Xystra, Charilaos Kostoulas, Urania Giougli, Konstantinos Pantos, Mara Simopoulou, Ioannis Georgiou

**Affiliations:** 1 Centre for Human Reproduction, Genesis Athens Clinic, 14-16, Papanikoli, 15232 Athens, Greece; sfakianosc@yahoo.gr (K.S.); info@pantos.gr (K.P.); 2 Laboratory of Medical Genetics, Faculty of Medicine, School of Health Sciences, University of Ioannina, 45110 Ioannina, Greece; zikopoulos.athan@outloook.com (A.Z.); nseretis@yahoo.com (N.S.); chkostoulas@gmail.com (C.K.); inogiouglii99@gmail.com (U.G.); igeorgiou@uoi.gr (I.G.); 3 Obstetrics and Gynecology, Royal Cornwall Hospital, Treliske, Truro TR1 3LJ, UK; 4 Department of Physiology, Medical School, National and Kapodistrian University of Athens, 11527 Athens, Greece; sokratis-grigoriadis@hotmail.com (S.G.); vagmaziotis@gmail.com (E.M.); evixistra@gmail.com (P.X.); 5 Department of Obstetrics and Gynecology, Faculty of Medicine, School of Health Sciences, University of Thessaly, 41222 Larisa, Greece; ganif@uth.gr

**Keywords:** medically assisted reproduction, in vitro fertilization, one-carbon metabolism, infertility, methyl donors, methylation, imprinting

## Abstract

One-carbon (1-C) metabolic deficiency impairs homeostasis, driving disease development, including infertility. It is of importance to summarize the current evidence regarding the clinical utility of 1-C metabolism-related biomolecules and methyl donors, namely, folate, betaine, choline, vitamin B12, homocysteine (Hcy), and zinc, as potential biomarkers, dietary supplements, and culture media supplements in the context of medically assisted reproduction (MAR). A narrative review of the literature was conducted in the PubMed/Medline database. Diet, ageing, and the endocrine milieu of individuals affect both 1-C metabolism and fertility status. In vitro fertilization (IVF) techniques, and culture conditions in particular, have a direct impact on 1-C metabolic activity in gametes and embryos. Critical analysis indicated that zinc supplementation in cryopreservation media may be a promising approach to reducing oxidative damage, while female serum homocysteine levels may be employed as a possible biomarker for predicting IVF outcomes. Nonetheless, the level of evidence is low, and future studies are needed to verify these data. One-carbon metabolism-related processes, including redox defense and epigenetic regulation, may be compromised in IVF-derived embryos. The study of 1-C metabolism may lead the way towards improving MAR efficiency and safety and ensuring the lifelong health of MAR infants.

## 1. Introduction

One of the most crucial mechanisms for genomic stability maintenance is DNA’s epigenetic marking through the methylation–demethylation cycle [1,2]. The methylation patterns of gametogenesis, fertilization, and embryo development have been deciphered over the last decades [3]. Proper methylation is essential for the normal inheritance of the various genes and appropriate gene regulation and expression. This is mainly achieved through parental effect genes that regulate epigenetic reprogramming, by regulating DNA and histone methylation and gene imprinting [4]. Imprinted genes predetermine gene expression, consolidation of the parental genomes, successful embryo cleavage, and the developmental and morphological conformations up to the stage of implantation and beyond [5].

The molecular and biochemical mechanisms involved in epigenetic processes during gametogenesis and embryo development, including DNA and histone methylation, strongly depend on one-carbon metabolism (1-C) [6]. One-carbon metabolism includes a chain of interrelated metabolic processes taking place in unison to supply methyl groups for purine and pyrimidine methylation, biogenic amine, and protein synthesis, as well as for phospholipid metabolism and redox balance. These processes are essential for epigenetic regulation, which in turn is crucial for proper cellular homeostasis and function [7]. One-carbon metabolism comprises three main pathways, namely, the folate cycle, the methionine cycle, and the transsulfuration pathway. The proper function of 1-C metabolism is mainly based on the availability of specific nutrients serving as methyl donor precursors. These include methionine, choline, betaine, vitamin B12, and folate (B9/11). Methyl donor precursors are mainly supplied by diet, by supplements, or even from gut microbiota functionality [8]. In addition to the abovementioned significant 1-C metabolism substrates, several other metabolites play a crucial role in 1-C metabolism functionality. Homocysteine (Hcy) is a substrate of great importance and constitutes the most well-studied biomarker of 1-C metabolism’s proper functionality [9]. In addition, a great body of evidence suggests that the biochemical reactions of 1-C metabolism are mediated by zinc-dependent enzymatic processes, finally resulting in methionine’s conversion to methyl donor S-adenosylmethionine (SAM). The latter acts as a universal methyl donor required for key epigenetic processes, including DNA and histone methylation [10,11,12]. Translating this knowledge to the level of clinical research, it is suggested that the abovementioned components of 1-C metabolism may emerge as useful tools towards developing novel diagnostic and therapeutic strategies for 1-C metabolism-related pathologies, including neurodegenerative diseases, cancer, and infertility [13,14,15]. 

Focusing on the physiology of reproduction, dysregulation of the mechanisms that control methylation and epigenetic reprogramming during gametogenesis and pregnancy can lead to gamete dysfunction and impaired embryo development, resulting in infertility and other pathologies, including recurrent pregnancy loss (RPL), recurrent implantation failure (RIF), and placental dysfunction [16]. Furthermore, the clinical importance of proper epigenetic regulation during gametogenesis, early embryonic development, and pregnancy is clearly outlined in Barker’s hypothesis of the “Developmental Origins of Health and Disease” [17]. According to Barker’s hypothesis, the complex epigenetic mechanisms that regulate embryonic development during the periconceptional period, as well as during the early and late stages of pregnancy, influence the lifelong health of infants. These mechanisms, in turn, are dynamically influenced by multiple factors and conditions, including ageing, parental health, diet, and lifestyle, and especially by the environment where embryonic and fetal development takes place [6,18,19,20,21,22]. 

Considering the above mentioned, the role of 1-C metabolism in reproductive physiology should be highlighted, since it constitutes a significant part of the mechanisms controlling methylation and epigenetic reprogramming during gametogenesis and embryo development [23,24,25]. This is especially important in the context of medically assisted reproduction (MAR), where several factors associated with infertility and MAR techniques are reported to alter 1-C metabolism and hence epigenetic regulation in both gametes and embryos [26,27,28]. In the context of exploring the link between 1-C metabolism and reproductive failure, recent data demonstrate that several substrates and metabolites of 1-C metabolism may have significant properties as biomarkers, dietary supplements, or even as supplements to gamete and embryo culture media in the context of in vitro fertilization (IVF) [29,30,31]. The rationale of the present study is to highlight the need for MAR specialists to elaborate the possible clinical implications of 1-C metabolism research findings in a “bench to bedside” fashion. This may pave the way for the development of personalized and precise management strategies to improve fertility care and, most importantly, to improve in vitro culture conditions of gametes and human preimplantation embryos. The ultimate goal of these efforts is to ensure the lifelong health of the MAR infants [32,33].

The aim of this narrative review is to summarize and analyze the current evidence regarding the clinical utility of 1-C metabolism substrates and other related nutrients in the context of MAR. More specifically, this review aims to highlight the role of the most well-studied 1-C metabolism substrates, namely, folate, betaine, choline, vitamin B12, and Hcy, as well as the role of zinc as biomarkers, dietary supplements, and culture media supplements in the context of infertility management, with a focus on MAR and IVF. Ultimately, this study aims to map future scientific goals at the clinical research level that underpin the potential diagnostic and therapeutic value of 1-C metabolism-related substrates.

## 2. Methodology Employed for Study Selection

A comprehensive review of the literature was conducted using the PubMed/Medline database. The search strategy process included a combination of medical subject headings (MeSH) terms and keywords, including “methyl donor”; “one-carbon metabolism”; “assisted reproductive technology”; “ART”; “medically assisted reproduction”; “MAR”; “in vitro fertilization”; and “IVF”. With regard to the literature screening methodology and study selection criteria employed, only English full-length articles published in international peer-reviewed journals were included. Acknowledging that this narrative review aims to summarize data of clinical relevance regarding the substrates of 1-C metabolism in MAR and IVF, the study selection process was restricted to human subjects. Specifically, included studies investigated the possible role of folate, betaine, choline, vitamin B12, and Hcy, as well as the role of zinc as biomarkers, dietary supplements, and culture media supplements in the context of MAR and IVF. However, data from studies employing basic research methodology and/or research in animal models were also analyzed as part of the discussion that follows in the next chapters of the manuscript. Regarding eligibility criteria for the methodology of the included studies, no specific inclusion and exclusion criteria were employed. Retrospective and prospective observational and interventional studies and randomized controlled trials of relevant data were all considered eligible for inclusion. Comparable evidence from other narrative reviews, systematic reviews, and meta-analyses was also discussed. Finally, relevant data were retrieved by manual citation mining.

## 3. Implications of One-Carbon Metabolism in Medically Assisted Reproduction

Prior to discussing the clinical implications of 1-C metabolism in MAR, it is important to review the molecular and biochemical processes of 1-C metabolism and the pathophysiological mechanisms linking 1-C metabolism to infertility and IVF techniques.

### 3.1. Outline of One Carbon Metabolism

As outlined in the Introduction of this manuscript, 1-C metabolism involves a multifaceted network of biochemical processes operating across various compartments of eukaryotic cells, including the cytoplasm, nucleus, and mitochondria [34,35,36]. The fundamental function of these processes is to facilitate the production of 1-C units in the form of methenyl, formyl, and methyl donors. In turn, these 1-C units are essential substrates of significant cellular process including molecular biosynthesis, genomic maintenance via the regulation of nucleotide abundance, epigenetic control via the regulation of DNA, RNA, and histone methylation, as well as redox balance [15,37]. Regarding the regulation of 1-C metabolism, it is established that it depends on the bioavailability of specific dietary components, nutrients, and metabolites such as folate, betaine, choline, vitamin B12, homocysteine, as well as zinc [34,38,39]. These molecules act as substrates, co-factors, and coenzymes for several metabolic process occurring within three metabolic pathways, namely, the folate cycle, methionine cycle, and transsulfuration pathway, which in union comprise 1-C metabolism.

With regards to the role of the folate cycle, it principally mediates the de novo synthesis of purines and thymidylate as well as the remethylation of homocysteine to methionine. The basic substrates of the folate cycle are folate and its synthetic form, folic acid. Folates are significant methyl donors that transport and activate one-carbon moieties and constitute the starting point of the folate cycle [35,36,40,41]. Folates cannot be endogenously synthesized and thus diet is the only source of these important substrates. In the small intestine and via the actions of gut microbiota, folates are hydrolyzed to their monoglutamated forms, enabling absorption [42,43]. This reaction is catalyzed by two enzymes, namely, γ-glutamyl hydrolase (GGH) and glutamate carboxypeptidase II (GCPII). The folate monoglutamates are then converted to 5-methyltetrahydrolate (5-mTHF), which is the major circulating form of folate in the peripheral blood. Along with choline, 5-mTHF is one of the main 1-C donors of the methionine cycle, as described below [16,34,44,45,46,47]. 

Regarding folic acid, this synthetic form of folate is totally oxidized and in this form is biologically inactive. The entrance of folic acid to the folate cycle is mediated by the enzyme DHF reductase (DHFR), which catalyzes the reduction of folic acid to dihydrofolate (DHF) and then to tetrahydrofolate (THF), which is the main biological active form of folic acid [37,48,49,50]. Tetrahydrofolate is a key molecule in the folate cycle. This is because it can be converted to 5,10-methylenetetrahydrofolate (5,10-CH2-THF) by the B6-dependent enzyme serine hydroxymethyltransferase (SHMT1) and then to 5-mTHF by irreversible reduction catalyzed by the “key” B2- and zinc-dependent enzyme methylenetetrahydrofolate reductase (MTHFR). The folate cycle is completed by an enzymatic reaction catalyzed by the B12-dependent enzyme methionine synthase (MTR), which demethylates 5-mTHF to remethylate Hcy to methionine. This process donates a 1-C moiety from the folate cycle to the methionine cycle to convert Hcy to methionine, which is one of the primary outcomes of the folate cycle [15,35,51,52,53]. 

The other major contribution of the folate cycle is the production of essential substrates for nucleotide synthesis, which in turn is crucial for maintaining genomic stability and repairing DNA damage. Briefly, as previously described, THF can be converted to 5,10-CH2-THF via SHMT1. Then, via a series of biochemical reactions catalyzed by methylenetetrahydrofolate dehydrogenases (MTHFDs), 5,10-CH2-THF is converted to 10-formyl-tetrahdrofolate (10-f-THF). 10-f-THF is an important biomolecule because it acts as a 1-C donor for the synthesis of purine rings [54]. In addition, 5,10-CH2-THF can be converted to DHF via the catalyzed actions of thymidylate synthase (TYMS). This enzyme uses 5,10-CH2-THF as a substrate and converts molecules of deoxyuridine monophosphate (dUMP) to thymidine monophosphate (dTMP) by transferring 1-C and is oxidized to DHF. In turn, DHF is reduced to THF, completing this metabolic loop of the folate cycle, as previously described. This reaction is of paramount importance since the de novo synthesis of dTMP occurs exclusively via methylation of the C-5 of dUMP by TYMS [14,54,55,56]. 

In summary, the folate cycle is a complex network of biochemical reactions in which folate and folic acid are used as 1-C donors for methionine and nucleotide production. It is logical to assume that the folate cycle is an important part of the molecular mechanisms regulating genomic stability and epigenetic modifications. It is also important to note that both folate and folic acid cannot be synthesized endogenously and therefore the bioavailability of these essential factors is exclusively dependent on dietary intake. Insufficient intake of these factors can therefore lead to significant disruption of the mechanisms that regulate DNA integrity and stability and thus of cellular homeostasis.

The methionine cycle is less complex than the folate cycle but of equal biological importance. The “key” biochemical reaction of the methionine cycle is the adenylation of methionine to S-adenosylmethionine (SAM), catalyzed by methionine adenosyltransferase (MATI/III). This production is crucial because SAM serves as a universal methyl donor required for several epigenetic processes, including methylation of DNA, RNA, histones, and lipids. SAM transmethylation, leading to the donation of methyl groups in the abovementioned biomolecules, is catalyzed by a group of enzymes known as SAM-dependent methyltransferases [57,58,59,60,61]. SAM-dependent methyltransferases are categorized according to the type of reaction they catalyze, namely, DNA methyltransferases (DNMTs), RNA methyltransferases (METTL3 and METTL14), protein arginine N-methyltransferases (PRMTs), histone methyltransferases (HMTs), glycine N-methyltransferase (GNMT), and others [53,62,63,64,65,66,67]. The regulation of these crucial enzymes is very complex and has been described in detail elsewhere [34]. Regardless of the type of SAM-dependent methyltransferases, the reaction of SAM transmethylation leads to the production of S-adenosylhomocysteine (SAH). In turn, S-adenosyl-L-homocysteine hydrolase (AHCY) converts SAH to Hcy and adenosine. The methionine cycle is completed by the catalyzed action of MTR, which remethylates Hcy to methionine using 1-C moieties provided by the folate cycle [68]. In addition, methionine remethylation by Hcy is also mediated by another alternative pathway. In this pathway, the 1-C moiety required for Hcy remethylation and methionine production is provided by choline. Briefly, choline is converted to betaine via the catalytic action of choline dehydrogenase (CHDH). Betaine in turn donates a methyl group to Hcy, resulting in the formation of dimethylglycine (DMG) and methionine. In this case, the transmethylation of Hcy to methionine is catalyzed by the enzymatic action of a B6- and zinc-dependent enzyme known as betaine-homocysteine S-methyltransferase (BHMT) [69]. In summary, the biochemical processes of the methionine cycle are essential for proper epigenetic regulation throughout development. The epigenetic roles of the methionine cycle are primarily mediated by SAM, which acts as a universal methyl donor. The bioavailability of SAM is in turn closely linked to methionine homeostasis. Methionine sufficiency is influenced by both diet and the proper functioning of the folate cycle, which is also influenced by diet. In addition, Hcy metabolism can also significantly affect methionine bioavailability and thus methionine cycle functionality [70,71,72].

The transsulfuration pathway is the third basic part of 1-C metabolism and plays a central role in sulfur metabolism and redox balance. Briefly, the pathway involves biochemical reactions that catalyze the transfer of sulfur from Hcy to cysteine via cystathionine. Cysteine is the main product of the transsulfuration pathway. Interestingly, this pathway is the only biochemical pathway of cysteine biosynthesis and is therefore of paramount importance for several cellular processes including protein synthesis and redox defense [73]. This is because, in addition to being a non-essential amino acid, cysteine is an important source of sulfur in human metabolism and has significant antioxidant properties [73,74,75]. In turn, cysteine serves as a substrate for two alternative biochemical pathways driving the synthesis of glutathione and taurine, respectively. Both glutathione and taurine also have significant antioxidant properties [76,77]. Regarding the mechanisms regulating transsulfuration pathway activity, these are strongly associated with the bioavailability of folate and methionine. When folate and methionine levels are adequate, indicating proper functioning of the folate and methionine cycles, approximately 50% of Hcy is irreversibly transsulfurated to cystathionine and cysteine [78,79,80]. These enzymatic reactions are catalyzed by cystathionine ß-synthase (CBS), a B6- and zinc-dependent enzyme, and cystathionine gamma-lyase (CTH), respectively. An observation of great importance is that these biochemical processes leading to cysteine synthesis are orchestrated by SAM. Upon folate and methionine sufficiency, SAM levels are increased and at this stage, SAM acts as an allosteric activator of CBS and inhibitor of MTHFR, regulating both the folate cycle and the transsulfuration pathway [34,81].

**Figure 1:**
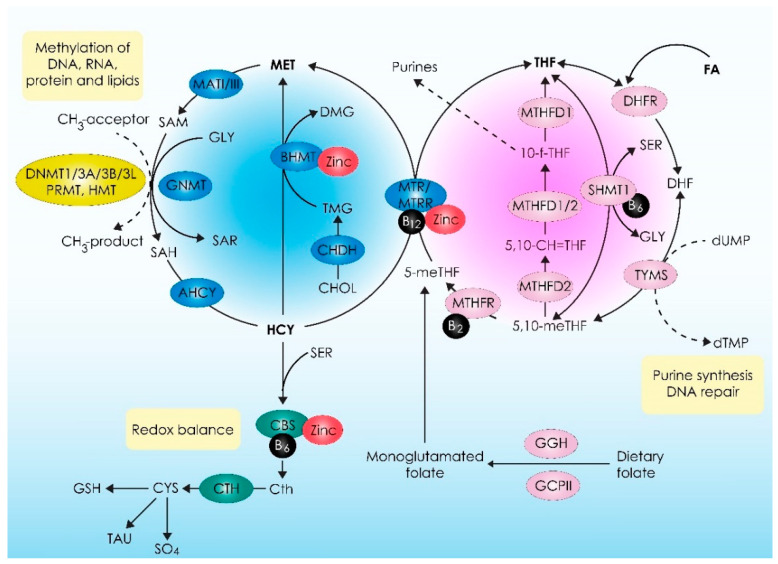
Outline of One-Carbon Metabolism and its related metabolic pathways. DHFR: dihydrofolate reductase; DHF: dihydrofolate; TYMS: thymidylate synthase; 5,10-meTHF: 5,10-methylene-tetrahydrofolate; MTHFR: 5,10-methylenetetrahydrofolate reductase; 5-meTHF: 5-methylenetetrahydrofolate; B12: vitamin B12; B2: vitamin B2; B6, vitamin B6; MTR: methionine synthase; MTRR: methionine synthase reductase; THF: tetrahydrofolate; MTHFD: methylenetetrahydrofolate dehydrogenases; 10-f-THF: 10-formyl-tetrahydrofolate; MTHFD1/2: methylenetetrahydrofolate dehydrogenase; 5,10-CH = THF: 5,10-methenyl-tetrahydrofolate; SHMT: serine hydroxymethyltransferase; SER: serine; GLY: glycine; FA: folic acid; dUMP: deoxyuridine monophosphate; dTMP: thymidine monophosphate; MET: methionine; MATI/III: methionine adenosyltransferase; SAM: S-adenosylmethionine; SAH: S-adenosylhomocysteine; GNMT: glycine N-methyltransferase; SAR: sarcosine; AHCY: S-adenosyl-l-homocysteine hydrolase; HCY: homocysteine; CHOL: choline; CHDH: choline dehydrogenase; TMG: trimethylglycine/betaine; BHMT: betaine-homocysteine S-methyltransferase; DMG: dimethylglycine; DNMT1/3A/3B/3L: de novo and maintenance DNA methyltransferases; HMT: histone methyltransferase; PRMT: protein arginine methyltransferase; CBS: cystathionine ß-synthase; Cth: cystathionine; CTH: cystathionine γ-lyase; GSH: glutathione; TAU: taurine; SO4: sulphate; GGH: γ-glutamyl hydrolase; GCPII: glutamate carboxypeptidase.

The complex biochemical reactions of 1-C metabolism as well as the basic substrates, enzymes, and co-factors are graphically presented in Figure 1. In conclusion, 1-C metabolism represents a complex network of tightly interconnected cycles and pathways and is an essential part of the biochemical mechanisms regulating several cellular processes, including epigenetic reprogramming, genome stability, metabolic regulation, and redox defense. This is because several important biomolecules with pleiotropic effects are exclusively synthesized via 1-C metabolism, including nucleotides, amino acids, and methyl donors. Due to the pleiotropic nature of its metabolites, 1-C metabolism affects several other metabolic pathways of great importance such as the propionate pathway, the polyamine pathway, the phosphatidylcholine pathway, the pathways for nucleotide biosynthesis, and several others [34]. Focusing on the regulation of 1-C metabolism, diet is a parameter of critical importance, as several substrates of 1-C metabolism are not endogenously synthesized, such as folate and folic acid [82,83,84,85]. In addition, several other biomolecules that are crucial for proper enzymatic functionality should also be provided by diet, including vitamins (e.g., vitamin B12) and trace elements (e.g., zinc) [86,87,88]. Apart from diet, endocrinological status also has a significant effect on the functionality of 1-C metabolism, and this particularly applies to sex steroid hormones [89,90,91,92]. Environmental pollutants and endocrine disruptors can also significantly impact 1-C metabolism [93,94,95,96,97]. With regard to the clinical implications of 1-C metabolism and considering the above mentioned, it is logical to assume that the dysregulation of 1-C metabolism could lead to several pathological conditions and vice versa [15]. This is evidenced by the fact that interventions targeting 1-C metabolism have been proposed as possible therapeutic strategies for several pathologies and developmental abnormalities and moreover several substrates of 1-C metabolism are used as biomarkers for monitoring general health status and disease progression [14,98,99]. More specifically, blood biomarkers such as methionine and folate levels, the SAM:SAH ratio, and plasma Hcy levels are used to clinically assess 1-C metabolic activity and the global methylation status of the organism [100,101,102,103]. Focusing on reproductive physiology, it is widely accepted that proper parental 1-C metabolic activity and regulation is required, particularly during the preconception period. Alterations in 1-C metabolism can significantly affect the epigenome and metabolic phenotype of the offspring, leading to developmental abnormalities and lifelong pathological conditions [6,34,104,105]. However, the pathophysiological mechanisms linking 1-C metabolism to infertility and MAR are hitherto not well understood.

### 3.2. One-Carbon Metabolism, Infertility, and Medically Assisted Reproduction 

According to the World Health Organization (WHO), infertility is a medical condition affecting both male and female reproductive systems. Infertility is defined as failure to achieve pregnancy by natural conception after 12 months or more of regular unprotected intercourse [106]. Global surveys estimate that between 48.5 and 72.4 million couples are infertile [107]. This means that the prevalence of infertility among couples of reproductive age is between 12.6% and 17.5% worldwide. The increasing prevalence of infertility highlights the urgent need to improve our understanding of the pathophysiological mechanisms through which the reproductive dynamic is compromised, with a view to improving reproductive health care in an era of precision and personalized medicine [108]. 

Regarding infertility etiology, male factor infertility accounts for 30% of infertility cases as the sole factor, while female factor infertility affects 30% of cases. Approximately 20% of infertile couples are affected by both male and female factor infertility [109]. Unfortunately, it is estimated that in approximately 15% to 30% of infertile couples, a standard fertility evaluation will not identify an abnormality in either partner, making up the pool of unexplained infertility cases [110]. Regarding the origins of infertility, several anatomical, congenital, acquired, genetic, and endocrine conditions can alter reproductive system functionality. Aging, in both men and women, also has a significant effect on an individual’s reproductive capacity [111,112,113]. In addition, several studies show that environmental and lifestyle parameters can also significantly affect the physiology of the reproductive system, leading to infertility [114,115]. 

Focusing on infertility management, several strategies have been developed over the years to effectively address infertility issues, including surgical and pharmaceutical approaches, ovulation induction, intrauterine insemination (IUI), and IVF. In vitro fertilization is the most widely used MAR technique, encompassing a variety of different strategies and laboratory protocols for embryo and gamete in vitro manipulation and culture [109]. When assessing the efficiency of the different MAR approaches based on the reproductive outcome, the most effective management strategy is IVF [116]. This explains the widespread use of IVF as the optimal strategy for managing a variety of different cases, considering both male and female factor infertility. However, when assessing MAR techniques according to the level of invasiveness in comparison to natural conception, IVF is the most invasive MAR procedure [117]. Acknowledging the invasive nature of IVF, substantial concerns have been raised regarding the impact of IVF protocols on the genomic stability and epigenetic regulation of gametes and embryos [118,119,120,121,122]. This holds significant importance, considering the available evidence supporting the hypothesis that epigenetic alterations during the early stages of embryo development may result in numerous pathological conditions during the later stages of life, including neurodevelopmental defects and endocrinological and malignant conditions [123].

To better understand the impact of IVF on the lifelong health of MAR infants, it is crucial to identify how infertility and IVF could alter the molecular mechanism regulating the epigenetic status of the gametes and preimplantation embryos. As analyzed previously, 1-C metabolism, its substrates, metabolites, enzymes, and co-factors are vital to several cellular processes that regulate the epigenetic mechanism throughout development. Therefore, it is important to investigate the possible mechanisms via which infertility and IVF impact 1-C metabolism functionality. This knowledge may be useful towards improving infertility management and IVF procedures, ultimately ensuring the lifelong health of infants born through MAR techniques.

To address this point of interest, it is important to highlight the possible factors that influence 1-C metabolism regulation in the context of infertility management. This is a very complex undertaking, considering the multifactorial nature of infertility pathogenesis, as well as the different methods and protocols used during IVF treatment, which adds another level of complexity. Considering the above, and prior to discussing the clinical significance of 1-C metabolism and its substrates on MAR, it would be beneficial to present data indicating how 1-C metabolism and epigenetics are influenced by several critical parameters associated with infertility management. These parameters include the etiology of infertility and the endocrinological status of the individuals, the fertility drugs used for ovarian stimulation in a typical IVF cycle, the insemination protocols used for IVF, and the in vitro culture conditions of gametes and preimplantation embryos. These parameters are considered critical because they can alter the epigenetic status of the gametes, and subsequently the epigenetic status of the resulting embryos, through several possible mechanisms. Focusing on the relationship between 1-C metabolism and infertility etiology, it is known that several underlying pathologies that affect the reproductive dynamic in both men and women also significantly affect 1-C metabolism. In turn, pathological conditions associated with 1-C metabolism dysfunction can affect the functionality of the reproductive system in both males and females [25,104,105,124,125,126]. 

More specifically, ageing is acknowledged as one of the most important determinants of both the male and female reproductive dynamic [111,127]. Regarding female fertility, as maternal age increases both the quantity and quality of oocytes, which are significantly impaired. This is specifically noted for women over the age of 35, a group widely known as the advanced maternal age (AMA) group. Several molecular mechanisms mediate the detrimental effects of ageing on oocyte competence. These include compromised hormonal status, alterations in cellular metabolism and mitochondria functionality, errors in chromosome segregation due to spindle instability and cohesion dysfunction, errors in the expression of maternal transcripts, oxidative stress, and defective epigenetic reprogramming during oocyte maturation [128]. Consequently, the compromised oocyte quality directly affects both the quality and the developmental dynamic of the derived embryos, leading to adverse obstetrical and perinatal outcomes, including infertility, RPL, preterm birth, preeclampsia, and intrauterine growth restriction [129,130]. Focusing on oocyte epigenetic alterations as maternal age increases, there is strong evidence suggesting that there is an age-dependent decline in the expression levels of several genes encoding DNMTs, including *DNMT1*, *DNMT3A*, *DNMT3B*, and *DNMT3L*, during oocyte maturation. These alterations impact the genome-wide DNA methylation pattern in the oocytes and subsequent embryos. This is crucial, because alterations in the oocyte epigenetic milieu can potentially be inherited by the embryo, affecting the proper functionality of the new organism [131,132,133]. Even though the exact impact of aging on human oocyte methylome is yet poorly understood, data indicate an abnormal expression of maternally inherited imprinted genes in oocytes of AMA patients, such as the gene encoding the tumor protein 73 (TP73) [134,135,136]. In addition, in a recently published study, where a comprehensive lipidomic and metabolomic analysis was performed in human immature oocytes and cumulus cells, a strong association between oocyte aging, oxidative damage, and mitochondrial dysfunction was observed. Analysis demonstrated a shift in the glutathione-to-oxiglutathione ratio and depletion of phospholipids. Interestingly, authors reported a significant age-related decrease in the total abundance of phosphatidylcholine, which is well known to be affected by 1-C metabolism, indicating its dysfunction [137]. These findings support the hypothesis that as maternal age increases, ovarian senescence impacts oocyte metabolic regulation, leading to 1-C metabolism dysfunction. Subsequently, the mechanisms connecting 1-C metabolism with the epigenetic regulation of the female germ line cells are compromised, leading to infertility as well as to adverse obstetrical and perinatal outcomes.

The impact of ageing on male reproductive capacity is also significant, with data supporting that advanced paternal age (APA) is associated with infertility, adverse pregnancy, obstetric and perinatal outcomes, and alterations in the lifelong health of offspring [138,139,140]. Despite these alarming findings, the role of paternal ageing when assessing the reproductive potential of a couple is underestimated by the national guidelines, and the assessment is often less comprehensive than that for the female partner [141,142]. This is mainly attributed to the fact that in contrast to oogenesis, spermatogenesis in mammals occurs throughout the lifespan of the male organism. However, as paternal age increases, spermatogenesis is negatively affected at several levels, including organ- and tissue-specific, cellular, and molecular levels. More specifically, age-related physiological events affect the proper functioning of the epididymis, prostate, seminal vesicles, and ultimately the testes. The hypothalamic–pituitary–testicular axis is also impaired with advancing paternal age, leading to impaired hormonal regulation and consequently impaired spermatogenesis [143,144]. As a result, a significant decline in the quantitative and qualitative parameters of the semen is observed, especially after the age of 40 [145]. In conclusion, APA is associated with impaired semen analysis parameters, including decreased spermatozoa concentration, motility, and vitality, as well as impaired spermatozoa morphology [113]. 

Focusing on the molecular level, several recently published studies indicate that APA is associated with impaired spermatozoa quality associated with de novo mutations, DNA instability and fragmentation, impaired spermatozoa metabolism, extensive oxidative damage due to ROS production, and finally abnormal epigenetic regulation [137,144,146,147,148,149]. Considering the age-dependent changes in the spermatozoa epigenome, there is evidence suggesting that this is mainly attributed to impaired expression and regulation of small non-coding RNAs, impaired histone modification including abnormal protamination, and, finally, impaired DNA methylation, leading to abnormally hypermethylated and hypomethylated DNA regions and consequently to abnormal genomic imprinting [144,150,151,152,153]. Interestingly, studies performed Gene Ontology analysis indicate that the previously described age-dependent epigenetic modifications on spermatozoa target gene pathways that regulate proper embryo development, neurodevelopment, growth, and metabolic function in offspring [152]. These data are important because they may provide a molecular explanation on the observed associations between APA and reduced natural fertility rates; reduced fertilization rates; reduced blastocyst formation rates; increased risk of MAR failure; increased risk of pregnancy and obstetric adverse outcomes, including preterm delivery, gestational diabetes, and neonatal seizures; increased risk of birth defects, including cleft lip, diaphragmatic hernia, right ventricular outflow tract obstruction, pulmonary stenosis, and increased risk of pediatric malignancies; and, finally, increased risk of psychological and neurodevelopmental disorders [140,154,155]. Future prospective and well-designed studies are needed in order to develop valid and accurate parental epigenetic clocks able to predict the exact impact of age-dependent epigenetic modifications on reproductive outcome as well as on the lifelong health of future generations. 

The mechanisms driving these age-related epigenetic changes, and, in particular, the role of 1-C metabolism, is poorly understood. However, it is well established that 1-C metabolism is crucial for proper spermatogenesis and spermiogenesis throughout the reproductive life of males [25]. Considering the evidence from studies investigating the role of 1-C metabolism in health and disease as well as the role of 1-C metabolism in ageing processes in other organ systems, we can hypothesize that as paternal age increases, 1-C metabolism in the testes and ancillary reproductive organs is compromised, resulting in abnormal spermatogenesis and spermiogenesis [15,156]. This hypothesis is supported by data suggesting impaired folate cycle function in ageing processes. As highlighted in Section 3.1, folate cycle is important for maintaining genomic stability and regulating epigenetic modifications, both of which are impaired in spermatozoa of APA individuals [41]. Therefore, studying the exact role of 1-C metabolism in the ageing processes of the male reproductive system is crucial not only for translational research, but also for daily clinical practice, given the ever-increasing trend of parenthood at an advanced age.

In addition to ageing, and to elaborate on the direct relationship between 1-C metabolism and infertility, it should be noted that dietary and/or genetic alterations leading to 1-C metabolism dysfunction can also cause infertility in both men and women [104,157,158,159]. In the context of IVF management, these individuals tend to present with compromised gamete quality, resulting in impaired embryo development and an increased risk of IVF failure. In 2019, Constance E. Clare and colleagues published a comprehensive literature review reporting that multiple variants of more than 30 genes encoding enzymes of 1-C metabolism are associated with infertility and adverse reproductive outcomes. The data presented highlight that the proper function of 1-C metabolism is critical for the proper production and maturation of gametes, for successful fertilization, for proper embryo development and implantation, for the maintenance of pregnancy and proper fetal development, and ultimately for the maintenance of lifelong infant health [34]. This is underlined by the fact that many of these variants are associated with both male and female infertility, impaired spermatozoa and oocyte quality, IVF failure, and complications related to pregnancy, fetal development, and labor, including RPL, preeclampsia, preterm delivery, low birth weight, and fetal death. Finally, impaired neonatal and pediatric outcomes have also been reported in cases of parental 1-C metabolic dysfunction, including neonatal hyperhomocysteinemia, neural tube defects, Kleefstra syndrome, congenital heart disease, Mudd’s disease, non-alcoholic fatty liver disease and others [34]. 

The most well-studied genetic variants related to 1-C metabolism are those detected in the gene encoding MTHFR. To date, more than 20 MTHFR polymorphisms have been described. Among these polymorphisms, MTHFR C677T is the most common and results in severely reduced MTHFR activity in vitro [160]. As previously described, MTHFR is a “key” enzyme that is critical for the proper functioning of the folate cycle, which in turn is critical for the conversion of Hcy to methionine. When MTHFR is dysfunctional, the methionine cycle is impaired, leading to epigenetic changes in gametes and subsequently in embryos. In addition, Hcy levels are elevated, leading to hyperhomocysteinemia, which is associated with infertility and adverse reproductive and neonatal outcomes, including small for gestational age (SGA) neonates, preeclampsia, and neural tube defects [161]. Clinical data support the above mechanisms. Several reports indicate that MTHFR polymorphisms are associated with reduced ovarian reserve, premature ovarian failure, impaired oocyte quality, impaired embryo development, embryo aneuploidy, RPL, and RIF [20,160,162,163,164]. However, there are conflicting data regarding the exact effect of MTHFR polymorphisms on clinical pregnancy and live birth rates [165]. In conclusion, future studies are needed to provide robust data on how and to what extent 1-C metabolism dysfunction affects reproductive dynamics. This is crucial not only for the timely diagnosis of underlying pathologies leading to infertility, but also for the development of individualized management strategies to improve impaired metabolism prior to IVF, such as dietary interventions. This may pave the way to reducing the time to pregnancy, increasing IVF success rates, ultimately preventing adverse pregnancy and obstetric outcomes, and ensuring lifelong health of infants.

In our efforts to better understand the intriguing mechanisms linking 1-C metabolism, infertility, and IVF, it is important to highlight that the endocrine milieu affects both 1-C metabolism and fertility status. Interestingly, several data support that genes involved in the regulation and function of 1-C metabolism and other related pathways are equipped with sex steroid hormone response elements [166,167,168]. In practice, this means that sex steroid hormones, including estrogens, progesterone, and androgens, directly or indirectly influence the proper regulation of 1-C metabolism and other important pathways, including the polyamine and phosphatidylcholinesterase pathways [89,91,169,170,171]. Taken together, existing evidence suggests that alterations in the proper regulation of sex steroid hormone production, synthesis, and secretion affect the proper functioning of 1-C metabolism, resulting in impaired epigenetic regulation and oxidative stress. The endocrine milieu of individuals is significantly influenced by age, internal and external factors, and, most importantly, by pathophysiological conditions and morbidities associated with endocrine system dysregulation [172,173]. For example, in women, estrogen and progesterone levels normally fluctuate during the menstrual cycle due to the cyclic regulation of the hypothalamic–pituitary–ovarian axis (HPO). Levels of sex steroid hormones also vary between women throughout their lives, namely, between pre-pubertal, pubertal, pre-menopausal, and menopausal states. For example, estradiol induces the expression of the phosphatidylethanolamine N-methyltransferase (PEMT) gene, suggesting that pre-menopausal women require less choline than postmenopausal women [90,92]. Similarly, changes in androgen levels, particularly testosterone, are observed with increasing age in men [174]. To conclude, aside from genetic background and diet, the endocrine milieu also plays crucial role in 1-C metabolism regulation and function, subsequently affecting nucleotide synthesis, DNA methylation, and oxidative stress regulation. 

The above mechanisms linking the endocrine milieu, and sex steroid hormones in particular, to 1-C metabolism should be considered in the context of MAR for two main reasons. The first reason is that endocrinopathies constitute common causes of infertility in both men and women. For example, the global prevalence of polycystic ovarian syndrome (PCOS) is estimated to be between 4% and 20% [175]. This means that infertility per se could affect steroidogenesis, leading to an imbalance in the production and secretion of sex steroid hormones. Given the important role of sex steroid hormones in 1-C metabolism function, it is logical to assume that infertility affects 1-C metabolism function, leading to epigenetic alterations and redox imbalance in gametes and embryos. 

The second reason is related to the use of controlled ovarian stimulation (COS) protocols in MAR and especially IVF, which aim to maximize the number of oocytes retrieved. These iatrogenic interventions have a direct impact on the endocrine milieu and thus on the 1-C metabolism [176]. Several COS protocols have been introduced into daily clinical practice, including the administration of exogenous gonadotropins, gonadotropin-releasing hormone (GnRH) analogues, and other reagents such as aromatase inhibitors [177]. Crucially, the exact effect of different COS protocols on 1-C metabolism remains unknown. To elaborate on that, data suggest that exogenous gonadotropin administration affects the levels of several biomarkers associated with the function of 1-C metabolism in both peripheral blood and follicular fluid samples, including B12 and Hcy levels [178,179]. It has also been reported that COS efficiency is influenced by 1-C metabolism, as low dietary levels of methionine, B12, and folate are associated with improved COS performance [16,180,181]. These interesting observations suggest that, on the one hand, COS impacts 1-C metabolism and, on the other hand, 1-C metabolism affects the efficiency of COS. This complex relationship is under investigation, and it is of paramount scientific interest, as the quality and developmental potential of oocytes and thus embryos is directly influenced by the ovarian, fallopian, and uterine microenvironment. 

In the scenario described above, where the condition of low dietary levels of methionine, B12, and folate is associated with improved COS performance, at the same time, Hcy levels are elevated. It is well established that elevated Hcy levels have detrimental effects on oocyte and embryo quality and developmental dynamics [6,16,29]. Several studies have provided data on the detrimental effects of high Hcy levels on follicular growth and oocyte competence. These studies indicate that in the absence of methionine, folate, B12, B6, and choline, antral follicle development and oocyte maturation are severely compromised, leading to impaired embryo competence and developmental arrest [31,182,183]. These phenomena are attributed to impaired regulation of methylation processes involving critical maternally imprinted genes such as *MEST* during folliculogenesis [184,185]. The direct effect of alterations in 1-C metabolism during oocyte maturation on embryo development can be explained by the fact that the 1-C metabolites and methyl donors required for proper embryo development until cleavage are provided by the fertilized oocyte and accumulated during oocyte maturation [23,186]. Several other reports support these data, indicating that COS affects the methylation status of several maternally inherited imprinted genes, including *PEG1*, *KCNQ1OT1*, and *ZACT*, and subsequently increases the risk of imprinting disorders in offspring [118,187,188,189,190]. The effect of COS on epigenetic regulation during oocyte maturation is likely to be mediated by changes in 1-C metabolism. In conclusion, the current rationale for developing novel COS protocols, which is to improve oocyte yield, should be shifted towards improving the safety of COS protocols to enhance oocyte quality and embryo developmental potential. 

Another level of complexity is added to the previously described chaotic mechanisms connecting 1-C metabolism with reproductive physiology, infertility management, and MAR when gamete and embryo in vitro handling and culture are co-evaluated. In humans it is challenging to extensively study the exact impact of IVF laboratory procedures on 1-C metabolism and thus on gamete and embryo epigenetic regulation. This is due to the co-existence of several other confounding factors, including infertility etiology, endocrine milieu, and iatrogenic interventions. However, data, mainly from studies in various animal models, support the hypothesis that IVF procedures, and especially in vitro culture, affect the epigenetic status of gametes and embryos, which is reflected in epigenetic alterations in offspring [191].

Focusing on in vitro culture systems, great advantages have been observed especially in the last decade, and today, high-quality, state-of-the-art, sophisticated culture systems, including time-lapse incubators, are available. These systems are designed to maintain stable conditions that allow for extensive uninterrupted embryo culture from the zygote to the blastocyst stage [192]. Great progress has also been noted in the efficiency of culture media. The available culture media can effectively support embryo development by providing the necessary micronutrients and metabolites, as well as a stable microenvironment with regulated redox balance [193,194]. Although available in vitro culture systems mimic in vivo conditions, it should be noted that during natural conception and from fertilization to implantation, both gametes and embryos are exposed to different microenvironments throughout the female reproductive system. The exact nature of these diverse microenvironments remains a “black box”, particularly in relation to human reproductive physiology [195]. Considering the above, it is logical to assume that in vitro embryo development differs significantly from in vivo development, and the effects of in vitro culture remain under investigation. The inadequacy of in vitro culture systems to fully mimic in vivo conditions has been linked to impaired epigenetic regulation of the embryo, resulting in epigenetic alterations in offspring [196]. 

The best studied paradigm is large offspring syndrome (LOS), first described in cattle and sheep. This syndrome is characterized by macrosomia, abdominal wall defects, organomegaly, and difficulty standing and suckling following parturition. Studies indicate that the incidence of LOS is significantly increased in in vitro-produced offspring [197,198,199]. In terms of pathophysiology, data suggest that LOS is caused by epigenetic changes leading to loss of imprinting (LOI) and expression of *IGF2R* and *M6P/IGF2R* [200]. Interestingly, the phenotypic characteristics of LOS syndrome in ruminants are similar to the phenotypic characteristics of Beck–Wiedemann syndrome (BWS) in humans, the incidence of which is also approximately three-to-nine times higher in MAR offspring compared to naturally conceived infants [201,202]. The BWS is also caused by alterations in two clusters of imprinted genes, namely, KCNQ1OT1 and CDKN1C [203]. It has been shown that the composition of the culture media, and particularly their metabolomic properties, drive these epigenetic alterations [34,118]. 

More specifically, reports indicate that different types of media differentially affect the methylation status of the preimplantation embryo genome. For example, differential expression of 951 genes involved in several cellular processes was observed in human embryos cultured in different culture media, namely, when cultured in G5 or human tubal fluid (HTF) [204]. These alterations may be attributed to 1-C metabolic dysfunction. This hypothesis is supported by evidence indicating that the incidence as well as the severity of LOS phenotype significantly increased in in vitro fertilization cases where embryos were cultured in the presence of serum. The addition of serum to the culture media was associated with increased intracellular SAM production in embryonic cells. This subsequently caused alterations in the SAM:SAH ratio in the resulting blastocysts, explaining the observed LOS-associated epigenetic dysfunctions due to impaired methylation regulation of imprinted genes [34,205,206]. In view of the above, the use of SAM as a supplement in culture media was investigated as a potential approach to improve epigenetic regulation in in vitro-cultured embryos. Interestingly, the results indicated that supplementation of SAM at high doses also caused extensive epigenetic alterations, leading to abnormal hypermethylation and hypomethylation of differentially methylated regions (DMRs). More specifically, the extensive presence of SAM caused hypomethylation in genes encoding factors involved in DNA demethylation processes, such as the BER pathway and *TET3*, and hypermethylation in genes encoding factors critical for DNA methylation, including *DNMT3B* [207]. These observations provide an exceptional paradigm of the plasticity that characterizes preimplantation embryos, which are equipped with unique adaptive mechanisms that enable them to compensate for any perturbation caused by factors in their microenvironment. However, although these adaptations are crucial for maintaining embryo viability, they are also the proximate cause of epigenetic changes that affect the lifelong health of MAR infants. In conclusion, there is evidence to suggest that the composition of culture media, particularly in terms of 1-C metabolites and methyl donors, has a significant impact on the methylation status of preimplantation embryos and subsequently on the epigenetic milieu of offspring [33]. This may be the origin of the observed increased incidence of epigenetic disorders such as BWS syndrome in MAR infants. Future prospective well-designed studies are needed to verify these observations, since recent published data argue against this hypothesis [208,209]. Moreover, there is a clear need for future studies at both basic and translational levels to further elucidate the long-term consequences of in vitro culture conditions on health and disease.

In summary, an intricate network of molecular, cellular, and physiological processes links 1-C metabolism, infertility, and MAR. There is considerable evidence that parental 1-C metabolism disorders are associated with impaired fertility status and vice versa. In addition, diet, ageing, various internal and external biological factors, and the endocrine milieu of individuals affect both 1-C metabolism and fertility status, and consequently the resulting gametes and embryos. In addition, IVF techniques and especially culture conditions have a direct impact on 1-C metabolic activity in gametes and embryos. As a result, 1-C metabolism-related processes, including redox defense and epigenetic regulation, may be compromised in IVF-derived embryos. Preimplantation embryos are endowed with unique homeostatic mechanisms that allow for modification of their epigenetic status to compensate for these perturbations. However, the precise impact of these epigenetic adaptations on the lifelong health of MAR infants is still under investigation.

## 4. One-Carbon Metabolism and Medically Assisted Reproduction: Clinical Implications

**Figure 2:**
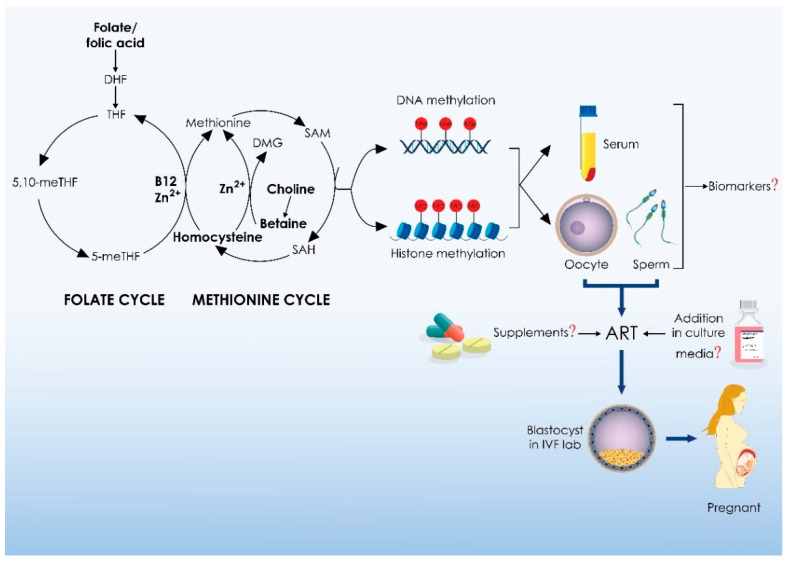
**Clinical implications of 1-C metabolism and methyl donors in medically assisted reproduction.** DHF: dihydrofolate; THF: tetrahydrofolate; 5,10-meTHF: 5,10-methylenetetrahydrofolate; 5-meTHF: 5-methylenetetrahydrofolate; B21: vitamin B12; Zn^2+^: zinc; DMG: dimethylglycine; SAM: S-adenosylmethionine; SAH: S-adenosylhomocysteine; ART: assisted reproductive technology; IVF: in vitro fertilization.

A detailed analysis of 1-C metabolism and a comprehensive review of the effects of 1-C metabolism on MAR is provided above, indicating that 1-C metabolism dysfunction is associated with infertility, MAR outcomes, and gamete and embryo competence. Recognizing the importance of 1-C metabolism in reproductive medicine, studies have focused on providing evidence regarding the clinical utility of 1-C metabolites and trace elements on MAR as biomarkers, dietary supplements, and supplements in gamete and embryo culture media, as shown in Figure 2. More specifically, MAR studies providing evidence with regards to the clinical utility of zinc, folate, B12, choline, betaine, and Hcy are comprehensively analyzed herein. 

### 4.1. Clinical Implications of Zinc in Medically Assisted Reproduction

As previously described, zinc is an important dietary complement, mediating crucial biochemical reactions in several 1-C metabolic pathways. Considering the role of 1-C metabolism in the proper function of the reproductive system in both females and males, zinc has been proposed as a biomarker and dietary supplement for assessing and improving the reproductive status of individuals. Moreover, acknowledging the role of 1-C metabolism in regulating redox balance, zinc supplementation has also been proposed as an approach to reducing oxidative stress damage on cryopreserved gametes. The evidence is summarized in Table 1.

Considering female reproduction, recent studies indicate that zinc is necessary for the proper regulation of folliculogenesis and oocyte maturation [231]. Although the exact role of zinc in oocyte competence has not yet been fully elucidated, several published data have shed light on this issue. In women, zinc levels have been mainly evaluated as a biomarker of oocyte competence and pregnancy outcomes. Two studies reported results on serum zinc levels, providing conflicting data, as the one reported no statistically significant impact of zinc on reproductive outcomes [215], and the other reported a positive correlation between serum zinc levels and clinical pregnancy rates [213]. Hair levels of zinc, as evaluated by one study, provided a statistically significant correlation with oocyte yield; however, they did not find any correlation with serum levels [225]. Regarding follicular fluid, safe conclusions cannot be reached, as one study observed no statistically significant association [216], while another one observed negative correlation of zinc levels with fertilization rate [218]. Another study observed higher follicular fluid zinc levels in women with endometriosis compared to tubal factor infertility [222]. Moreover, in women with endometriosis, the study reported that zinc levels were associated with the probability of clinical pregnancy, while no statistically significant association was observed in women with tubal factor infertility [222]. A single study evaluated urine zinc levels in women and reported a positive correlation with the number of available embryos obtained [218]. Summarizing the current evidence, no safe conclusions can be made with regard to the clinical usefulness of zinc as a biomarker for assessing female reproductive potential and MAR outcomes, since heterogenous and conflicting data exist. This heterogeneity may be attributed to the fact that several different approaches have been applied for evaluating zinc levels in different biological samples, including serum and follicular fluid. Moreover, no consistency is observed among the existing studies with regard to the studied populations. However, there is strong evidence indicating that zinc is an essential micronutrient crucial for the proper functioning of the female reproductive system, and thus future studies are needed to unveil the clinical implications of zinc in MAR.

In the literature, the necessity of zinc is mainly supported through animal studies. Oocyte chromatin methylation and preimplantation growth were disrupted when animals were provided with a zinc-deficient diet (ZDD) before ovulation [231]. Zinc-deficient oocytes demonstrated a decrease in histone H3K4 trimethylation (H3K4me3) as well as in global DNA methylation [231]. Supplementation of SAM in in vitro maturation (IVM) media restored H3K4me3 and increased the IVF success rate from 17% to 43% [231]. The above experiments highlighted that the oocyte maturation from the germinal vehicle (GV) to metaphase II (MII) stage is highly dependent upon zinc availability, which, consequently, may also affect IVF outcomes [231]. In addition to this, the use of zinc in the maturation medium for oocytes showed an increase in maturation, cleavage, and blastocyst rates [232]. A positive correlation was observed between follicular fluid zinc levels and oocyte quantity and quality, while a threshold zinc concentration above 35 μg/dL was assessed as crucial for better embryo quality [233]. Although the positive role of zinc is established, it appears that the impact of zinc is also concentration-dependent [234]. That means that zinc levels above the normal limits exert a negative effect on oocyte maturation and therefore oocyte competence and quality [234]. It could be hypothesized that zinc levels act similarly to a reverse U-curve with only levels between specific cut-off values being beneficial, while beyond them, zinc may be harmful, compromising oocyte competence. Recently, it was found that exaggerating intracellular zinc may result in oocyte meiosis disruption through the involvement of MTOC-associated proteins, which in turn results in spindle defects and altered epigenetic modifications [234]. 

In addition, oocyte activation, the process that takes place soon after fertilization and plays pivotal role in the pronuclear formation and embryo development, has also been linked with the presence of zinc. Apart from the central role of calcium in this process, it has been documented that reducing available bivalent zinc contents may provide new approaches to improve artificial oocyte activation [235]. Similarly, zinc status within the appropriate limits in cattle research has been studied, and it was observed that semen parameters were significantly improved, but without any positive effect on IVF outcome [236]. Zinc seems to play an active role during the fertilization process. The presence of zinc in the fertilization medium appears to have a beneficial effect on zona pellucida-binding spermatozoa, indicating indirectly that the presence of trace minerals is additionally essential for the completion of the fertilization process [237]. Accordingly, with oocyte activation, zinc spark, the release of zinc ions during calcium oscillations, was associated with embryo quality in a mouse model, suggesting that zinc can also serve and act as a cellular biomarker for embryo quality [238]. Despite the above findings in animal models, in humans, a follow-up study evaluating methylation patterns in the participants of the aforementioned RCT reported no significant alterations, despite that a number of trends were observed [239].

Regarding the male reproductive system, testosterone synthesis, sperm maturation, and testicular development have been associated with zinc levels [240]. It should be noted that zinc, besides regulating 1-C metabolism, may exert antioxidant effects. This may allow for zinc acting on various levels, which may account for the mechanism of action discussed in several studies included in this review. A total of five studies investigated the association between zinc levels and male fertility. Three of them evaluated the levels of zinc in seminal plasma while the remaining two evaluated serum zinc levels. One out of three studies reporting on seminal plasma levels of zinc reported that fertile men presented with higher zinc levels compared to infertile [226]. Moreover, one study reported that zinc levels may be positively correlated with sperm concentration and motility; howver, a negative correlation was observed with spermatozoa morphology [224]. The last study did not report any statistically significant association with semen parameters [229]. Regarding zinc serum levels, one study reported no statistically significant association between zinc levels and fertility status [210], while the other one reported higher levels of zinc in fertile men compared to those infertile [212]. 

Zinc supplementation in males was evaluated in a total of six studies, with a total of eight arms. Six studies evaluated supplementation of zinc solely, one arm of one study evaluated zinc with multiple other vitamins, and two studies evaluated the combination of zinc with folic acid. Results indicated that supplementation of zinc improves male fertility parameters, in terms of spermatozoa morphology and motility, and all studies reported a decrease in DNA fragmentation index (DFI). As observed in animal models, zinc, like other biological factors, exerts its action in a dose-dependent manner. The administration of low concentrations of zinc oxide did not induce any significant alteration in sperm motility and viability, while elevated levels resulted in reduced motility and viability, as were evident with the use of mitochondrial toxicity assay [241], indicating the role of various dietary supplements, including zinc, in male fertility [242]. This positive effect of zinc was previous reported following sperm incubation with zinc of samples originating from normozoospermic men and a asthenozoospermic patients [220]. It was demonstrated that incubation with zinc maintained spermatozoa motility in normozoospermic men and improved it significantly in asthenozoospermic patients, irrespective of the intracellular concentration of zinc [220]. 

In contrast, it has been observed that the presence of zinc decreases spermatozoa motility, while this decrease is restored after zinc elimination [243]. A hypothesis for this effect of increased spermatozoa motility after incubation with zinc is that it may be related to the lower zinc concentration used, compared to other previous studies. Regarding intracellular zinc, decreased levels throughout sperm epididymal transit leads to progressive and eventually hyperactivated motility attained during the sperm capacitation process [243,244]. In addition to this, through its interaction with the zinc sensing receptor (ZnR), also known as GPR39, extracellular zinc appears to have an impact on the related intracellular signaling pathways. The presence of the abovementioned receptor in spermatozoa tail and acrosome suggests that zinc may be involved in spermatozoa motility regulation and acrosomal exocytosis. Studies on animal models showed that bovine sperm acrosomal exocytosis is also stimulated by zinc as well as that bovine and human spermatozoa hyperactivation and progressive motility, were both mediated by GPR39 [243,244,245]. 

Moreover, mutations in the zinc finger MYND-type-containing 15 gene (*ZMYND15*) have been associated with non-obstructive azoospermia (NOA) and severe oligozoospermia [246]. The importance of zinc for spermatozoa function was postulated due to the higher—than those found in other tissues—levels in the seminal fluid [220,226]. Nevertheless, it is worth mentioning that reports on the effects of zinc on spermatozoa motility are conflicting, with recent studies reporting no correlation between zinc and spermatozoa motility or concentration [220,247]. Further evidence supports the notion that a micronutrient zinc diet reduced the presence of spermatozoa DNA damage both in animal models and infertile men through the activation of the endogenous antioxidant system [248]. Recently, micronutrient supplementation was shown to improve spermatozoa nuclear maturation as well as semen antioxidant capacity, resulting in reduced DNA damage and lipid peroxidation [248]. These findings seem promising; however, further studies are required prior to concluding on the effectiveness of zinc dietary supplementation as an adjuvant antioxidant therapy in infertile patients with or without inflammatory disorders and increased DFI. While zinc may not serve as a sensitive biomarker for assessing male fertility status, it seems that zinc supplementation improves semen parameters. However, in order to conclude on zinc’s possible therapeutic effects, further large, well-designed randomized controlled trials (RCTs) are required prior to offering zinc supplementation in daily clinical practice as a strategy to improve male reproductive capacity.

Considering the role of zinc as a culture media supplement, a total of three studies evaluated the use of zinc as a supplement in sperm cryopreservation media [217,219,223]. Two of them performed statistical comparisons, indicating that zinc supplementation was associated with higher chromatin integrity and higher progressive motility [219,223]. Regarding the third study, where statistical analysis was not performed, a similar trend was observed [217]. Sperm cryopreservation is widely employed in daily clinical practice; however, it may be associated with detrimental effects on the spermatozoa, as observed following thawing [243]. Indicatively, after thawing, spermatozoa present with lower fertilization capacity, reduced motility and viability, abnormal morphology, cell membrane damage, increased DNA fragmentation, and reduced mitochondrial activity [243]. Zinc supplementation in culture media improves spermatozoa performance after thawing by reducing the effects of potent oxidative agents, such as hydrogen peroxide, which increases SDF [249]. This notion is confirmed by a more recent study indicating the beneficial effects of zinc supplementation before cryopreservation on sperm viability and motility [217]. Once human spermatozoa were frozen in the presence of 50 mM of zinc, an increase in the proportion of progressive motility and the number of motile spermatozoa of 184% and 130%, respectively, was observed after thawing [217]. Similar results were obtained after the supplementation of 100 μg/mL zinc (NZn) and zinc oxide (NZnO). After supplementation, spermatozoa presented significantly higher total and progressive motility, mitochondrial activity, viability, and membrane integrity, and lower lipid peroxidation compared to control groups, indicating that zinc supplementation is an essential factor for restoring spermatozoa quality and competence [250].

In conclusion, analysis of microelements, such as zinc, in semen samples, seems to be a useful tool for evaluating sperm cryotolerance [251], also minimizing the freeze–thaw-induced damage to spermatozoa [219]. Although it has not yet been proven clinically, zinc seems to have beneficial effects on the maintenance of spermatozoa genomic and chromosomal stability, as well as on membrane integrity and cell morphology [223,243,252]. The addition of zinc micromolar concentrations to gamete media seems to improve spermatozoa activation and motility, which, in turn, is expected to benefit the IVF outcomes [243]. According to the data presented herein, the addition of zinc in sperm cryopreservation media seems to be a promising option. Further studies, and more specifically, RCTs, are required prior to concluding on the effect of possible zinc supplementation in gamete and embryo cryopreservation media.

### 4.2. Clinical Implications of Folate in Medically Assisted Reproduction

Folate is required for the proper function of 1-C metabolic pathways as well as for proper epigenetic regulation. It has been reported that folate sufficiency is crucial during periods of rapid cell growth and proliferation, such as germ cell maturation and embryo development, because it mediates several molecular processes involved in DNA, RNA, and protein synthesis [181]. In particular, folate level adequacy has been studied in relation to poor pregnancy outcomes and, more recently, to pathophysiological conditions affecting female reproduction [181]. There is evidence that insufficient folate levels are associated with abnormal DNA methylation and increased blood Hcy levels [253]. Although there is no high level of evidence regarding the role of folate, it was demonstrated that adequate folate levels may prevent neural tube defects [254]. A recent meta-analysis concluded that folate levels do not have a significant impact on IVF outcomes [255]. On the contrary, a more recent RCT reported that when elevated serum folate levels were combined with a lower Ca/Mg ratio, a significant negative correlation with IVF outcomes was established, indicating a significant impact of micro- and macronutrient insufficiency on IVF outcomes [256]. However, a clear relationship between folate levels and IVF outcomes has not been established yet [257]. The evidence is summarized in Table 2.

More specifically, a total of seven studies reported results on the supplementation of folate, alone or in combination with other nutrients, in women undergoing IVF. It may be extrapolated that folate alone does not improve the IVF outcome; however, it may be beneficial when supplemented with other nutrients. In addition, it has been observed that certain gene mutations in women that affect the folate pathway may be associated with lower pregnancy rates. For folate to participate actively in 1-C metabolism, the MTHFR enzyme is required. It has been observed that mutations in *MTHFR* may be associated with poor IVF outcomes. Studies have reported conflicting results [160,266]; nonetheless, a more recent study reported that the combination of two mutations, namely, the C677T and A1298C mutations, decreases the number of MII oocytes retrieved and the number of embryos available for transfer [267]. Considering the results of a recent RCT, the FASZT trial, comparing supplementation of zinc combined with folic acid to a placebo group in males, no statistically significant difference was reported with regard to IVF outcomes [268]. 

In an animal experimental study, it was found that folate supplementation upregulated genes responsible for cumulus expansion, which is a characteristic of follicular growth, indicating a higher grade of maturation, that eventually led to the production of higher-quality blastocysts [269]. The possible cellular and molecular mechanism underlying this result may be the presence of folate receptor-1, which has been found to be vital for the competence of preimplantation embryos [269]. Similar results were obtained following the supplementation of folic acid during IVM of bovine oocytes. A positive correlation was observed in the animal model regarding blastocyst formation rate and blastocyst quality with folic acid levels, also preventing epigenetic errors in the offspring [270]. In addition, studies with animal models demonstrated the positive effect of folic acid supplementation on ethanol-induced developmental defects [271].

In conclusion, folate and folic acid are essential for the proper functioning of the reproductive system as well as for proper embryo development. Sufficient folic acid dietary consumption is of paramount importance both prior to and during pregnancy in order to maintain reproductive capability. However, caution is needed with regard to folic acid supplementation in the context of improving IVF outcomes in cases of sufficient folate and folic acid metabolism. This is mainly attributed to two reasons: The first reason is that there is insufficient evidence with regard to the role of folic acid supplantation for improving IVF outcomes and this may lead to inappropriate and time-consuming management. The second and most important reason is that there is evidence supporting that excessive folic acid supplementation on the grounds of folate sufficiency may be detrimental for embryo development, causing asthma and congenital malformations and might affect the long-term health outcomes of offspring [272]. In view of the above, the use of folic acid supplementation in everyday clinical practice in the context of MAR must be approached with extreme caution. An individualized approach is required in each case, and folic acid supplementation during periconception should only be initiated for folate insufficiency and for a specific duration. Future studies are needed to clarify the observed increased incidence of congenital malformations with excessive folic acid consumption, but it seems that folate homeostasis is highly sensitive, leading to developmental disorders involving both insufficient and excessive folate levels. It is likely that abnormal epigenetic regulation mediates these phenomena. 

### 4.3. Clinical Implications of Vitamin B12 in Medically Assisted Reproduction

The role of various vitamins has been investigated in the context of improving MAR outcomes. Among the variety of micronutrients that are required during pregnancy besides folate, vitamin B12 seems to be of significant importance in controlling and altering DNA methylation patterns by regulating the transfer of methyl groups through 1-C metabolism [273]. Particularly, vitamin B12 seems to play an essential role in the regulation of DNA methylation processes, exerting its action through participation in homocysteine metabolism [274]. When a methyl group is transferred to homocysteine to form methionine, vitamin B12 acts as a co-factor for the methionine synthase enzyme [274,275]. 

Investigating the possible association of folate and vitamin B12 levels with MAR outcome, it has been reported that women of the highest quartile of both serum folate and vitamin B12 presented with higher chances of live birth compared to women of the lowest quartile of the respective nutrients [276]. The two nutrients are thought to interact with each another, and their combined effect may have an impact on the quality of oocytes, embryo competence, and subsequently with MAR outcomes [276]. Recently, in the Environment and Reproductive Health (EARTH) study, it was shown that higher intake of folic acid, vitamin B12, and vitamin D was associated with an increased frequency of live birth in women undergoing MAR [277,278,279]. Similarly, in a cohort study of women undergoing IVF, vitamin D receptors appear to influence vitamin B12 and folic acid absorption where the body mass index appears to have an indirect effect through the interaction with vitamin B12 and folic acid [280]. In addition, data support the hypothesis that supplementation of the vitamin B complex, 5-methyltetrahydrofolate, vitamin B12, and vitamin B6, compared to folic acid supplementation alone seems to act more beneficially in terms of clinical pregnancy and live birth rate in women undergoing IVF treatment [281]. 

It seems that insufficient diet of folate, and vitamin B12, in combination with co-existing pathologies, such as autoimmunity and iron deficiency, which appear to require a higher intake of folate and vitamin B12, directly leads to nutritional-dependent infertility [282]. Data from animal studies indicated that vitamin B12 deficiency, independently of folic acid levels, affects the expression of crucial genes encoding 1-C metabolism enzymes [283]. Limited data hitherto suggest that vitamin B12 supplementation may exert a positive effect on IVF outcomes. Nonetheless, further studies are required to verify these conclusions. 

### 4.4. Clinical Implications of Choline in Medically Assisted Reproduction

As previously described in this review, choline holds a significant role in 1-C metabolism since it is one of the main 1-C donors of the methionine cycle. This underlies the importance of choline sufficiency during the periconception period as well as during embryo development [284]. Choline has been proposed as a possible marker in cases of RIF and PCOS [285,286]. There is evidence suggesting that high choline levels have been associated with successful pregnancy outcomes in both RIF and PCOS populations. In addition, the levels of its metabolic product, trimethylamine-N-oxide (TMAO), have been associated with poor fertilization rate and lower embryo quality, while choline per se provided no statistically significant association [287]. This is in agreement with a previous study presenting similar findings [288]. 

Choline seems to also be important regarding male fertility, as its levels have been observed to be lower in asthenozoospermic men [289]. Moreover, polymorphisms in the genes of choline dehydrogenase and phosphotransferase 1 have been associated with low sperm concentration and spermatozoa head defects, respectively [290,291]. 

Limited data exist regarding choline use as a supplement in IVF culture media. In the past, choline has been used as a supplement in cryoprotectants; however, only in the context of slow-freezing [292]. 

A great body of evidence underscores the significance of sufficient choline intake during pregnancy. During the prenatal period, choline is vital for numerous physiological processes, including membrane biosynthesis, tissue expansion, neurotransmission, and brain development [82,170,293,294]. Moreover, it has been reported that choline supplementation during pregnancy may mitigate the severity of fetal alcohol spectral disorders (FASDs) [295]. 

According to the above mentioned, it may be extrapolated that choline is crucial for reproductive system function as well as for proper embryo and fetal development. However, data regarding its impact on improving fertility outcomes in the context of MAR remains controversial.

### 4.5. Clinical Implications of Betaine in Medically Assisted Reproduction

Betaine is a metabolite of choline. The transmethylation process of betaine occurs mostly in mitochondria of the liver and kidney, as a part of a 1-C metabolism via the methionine cycle [296]. The role of betaine on reproductive system physiology has mainly been investigated in animal models. Only one study has evaluated supplementation of betaine in humans. When administered along with folic acid and other antioxidant supplements, betaine improved spermatozoa parameters, fertilization rate, and embryo quality in male infertility cases [297]. Since variations in the choline dehydrogenase gene, which impact the metabolism of betaine, affect male fertility, it may be worth further investigating if betaine could be proposed as a possible supplement for improving the reproductive dynamic in male infertility cases.

Focusing on the results of the animal studies, it has been reported that dietary betaine supplementation has an impact on various sulfur amino acids (SAAs) by increasing the available methionine and SAM. As a result, betaine acts as a methyl donor and appears to be essential for SAA metabolism [296]. Current findings demonstrate that betaine may alter the negative effects of ethanol by restoring global methylation levels in blastocysts [298]. Maternal excessive ethanol uptake may have detrimental effects on preimplantation embryo development [299]. Since DNA methylation plays a crucial role in the regulation of gene expression during embryogenesis and organogenesis, ethanol-associated alterations in fetal DNA methylation may contribute to the developmental abnormalities in fetal alcohol syndrome [300]. The direct consequence of this is the higher production of ROS, which interfere in 1-C metabolism [299]. 

Taking into account the aforementioned, in an animal study employing embryos cultured in a medium containing 1% ethanol, impaired blastocyst formation and, subsequently, compromised implantation potential were observed [298]. These detrimental effects on embryo development were nullified following supplementation of 50 μg/mL betaine into culture medium. Betaine supplementation reduced ROS production caused by ethanol and restored embryo developmental potential [298]. Live-born infants who were exposed to alcohol during pregnancy were found to have life-threatening congenital heart abnormalities. Studies in animal models observed that betaine-supplemented ethanol-exposed embryos presented with higher late-stage survival rates and fewer severe head and body abnormalities compared to the control group [301]. Additionally, betaine decreased the prevalence of late-stage heart abnormalities, suggesting that low-concentration betaine supplementation may mitigate FASD [301].

In conclusion, animal studies provide strong evidence indicating that betaine holds significant antioxidant properties able to improve embryo developmental capacity in cases of severe oxidative damage. Considering that several endogenous and exogenous factors induce oxidative damage in gametes and embryos during IVF, future studies in humans should focus on providing evidence with regards to the role of betaine as a supplement in IVF culture media. 

### 4.6. Clinical Implications of Homocysteine in Medically Assisted Reproduction

Regarding Hcy, studies reported an adverse effect on embryological parameters, as an inverse correlation between follicular fluid Hcy levels and oocyte and embryo quality was documented [302]. These findings underline the role of Hcy as a potential and useful candidate marker for fertilization potential and oocyte and embryo quality in patients undergoing IVF treatment [302]. During IVF, elevated homocysteine levels in follicular fluid are associated with varying degrees of oocyte immaturity and poor embryo quality [303]. Homocysteine accumulation can be attributed to either dietary or genetic folate deficiency, while unmetabolized Hcy through transportation to the blood causes hyperhomocysteinemia [181,304]. The latter has been also associated with numerous pathophysiological conditions during pregnancy. 

Maternal Hcy levels have been linked to an increased risk of miscarriage in women undergoing IVF and defective chorionic villous vascularization in women with RPL [26,181,305]. Additionally, in in vitro experimental studies, Hcy has been found to induce trophoblast apoptosis, as well as to reduce human chorionic gonadotropin secretion [306]. Folate supplementation increases serum folate levels and decreases Hcy levels in the cellular milieu of mature oocytes [181]. Additionally, in another study, Hcy levels were statistically significant different between pregnant and non-pregnant women, while all the other embryological parameters studied were comparable between the two groups [303]. The imbalance of homocysteine in the follicular fluid, which is the ambient microenvironment of oocytes, may cause abnormal oocyte development. In a more recent study, Hcy levels in follicular fluid were negatively associated with oocyte maturity, embryo quality, and pregnancy rates [307]. 

Although aging may indirectly contribute to higher Hcy follicular fluid levels, this is another underlying mechanism contributing to the decreased embryo quality and oocyte maturation observed in AMA cases undergoing IVF. In contrast, it was observed that pregnancy outcomes were comparable even if Hcy levels were positively associated with the total number of oocytes retrieved indicating that Hcy concentrations do not significantly affect MAR outcomes [179]. It should be mentioned that *MTHFR* mutations have been associated with higher serum Hcy levels, and their combination may provide more harmful effects compared to cases with higher Hcy levels albeit without the specific mutation [179]. 

Interestingly, Hcy has been proposed as a potential biomarker for assessing preimplantation embryo metabolic activity. Results provided by a study evaluating Hcy levels in spent culture media of cleavage-stage embryos reported a negative association between Hcy levels and pregnancy outcomes [308]. Particularly, the cut-off value of Hcy levels indicating optimal embryo quality and pregnancy success was found to be 3.53 μmol/L [308]. 

To summarize the current evidence, a total of five studies were identified to evaluate Hcy levels in serum and/or in follicular fluid in women undergoing IVF (Table 3). Most of them concluded that Hcy may be employed as a negative biomarker for predicting IVF outcomes, as a negative association with multiple reproductive outcomes has been observed. Further studies introducing an evidence-based cut-off value of Hcy levels, or possible ranges that correspond to different outcomes, are required. Following their completion, the evaluated predictive accuracy of Hcy should be thoroughly investigated in terms of sensitivity, specificity, positive and negative predictive value, and other related metrics prior to introducing Hcy assessment in daily clinical practice.

### 4.7. The Role of Metabolomics in the Era of Personalized and Precision Medicine in Assisted Reproduction

Considering the discussion and data analysis presented in the previous chapters, it is shown that infertility diagnosis and management, MAR efficiency, and, most importantly, the lifelong health of MAR infants are significantly associated with the metabolomic milieu of individuals. Using 1-C metabolism as an example, we have shown that there is a large body of evidence to support the importance of developing research activities to gain more knowledge to better understand the complex molecular and biochemical mechanisms linking metabolic dysregulation with male and female infertility and early embryo development. 

However, this is a very challenging goal to be achieved, given the intertwined nature of the numerous metabolic pathways that regulate cellular homeostasis and development, indicating that a holistic assessment is required to complete the “missing pieces” in optimal MAR management. To elaborate on this, it is important to study metabolism as a whole to develop precise and personalized diagnostic and therapeutic MAR management strategies. This can be achieved with novel and sophisticated systems capable of providing comprehensive and detailed metabolomic analysis in multiple biological samples, including follicular fluid, seminal plasma, and spent embryo culture media [313,314,315]. Several recently published studies demonstrate that metabolomic profiling may be useful in almost every step of MAR procedures, including diagnosis of the underlying pathology leading to infertility, design of appropriate treatment strategies, as well as selection of the embryo with optimal developmental dynamics [316]. This is of paramount importance, as all of the above contribute to improving the efficiency and safety of MAR procedures by reducing the time to achieve pregnancy, avoiding unnecessary interventions and, most importantly, avoiding adverse outcomes. 

In the context of metabolomics in MAR, two analytical methods are widely used to assess ovarian, testicular, and embryonic metabolism, namely, nuclear magnetic resonance (NMR) and mass spectrometry (MS). Both methods are well standardized and provide a detailed analysis of the metabolic profile at both the qualitative and quantitative level. Despite the advantages and disadvantages of these techniques, which are well known and characterized, both are considered as “gold standard” tools for the study of the metabolic characteristics of follicular fluid, seminal plasma, and spent culture media samples, emerging as cost-effective, accurate, and non-invasive approaches to uncovering and characterizing the role of metabolism in the development of infertility-related pathologies. In addition, both methods can be employed to make comparisons between the metabolic profile of different biological samples, such as follicular fluid/seminal plasma versus blood serum samples, allowing for the identification of possible biomarkers for assessing the metabolic profile of individuals in the context of infertility diagnosis [317,318]. In summary, the combinatorial use of both NMR and MS should be considered the “gold standard” approach for assessing the metabolic profile of individuals undergoing MAR. This combinatorial use provides an accurate, sensitive, and non-invasive approach to detecting metabolic dysregulation in the context of infertility management. As indicated in the following paragraphs, the use of NMR/MS has already provided new data that have enriched knowledge of the MAR specialist and significantly improved infertility diagnosis and management. 

Considering the use of metabolomic analysis via NMR/MS for the assessment of ovarian metabolism, several studies have been published in the last decade, indicating impaired ovarian metabolic regulation in populations with three major causes of female infertility, namely, endometriosis, PCOS, and diminished ovarian reserve (DOR) [313]. 

Regarding endometriosis, metabolomic analysis of follicular fluid samples from endometriosis patients undergoing IVF revealed abnormal levels of numerous metabolites associated with several different pathways. Specifically, ovarian levels of carbohydrates, lipids, and ketone bodies are significantly disturbed in patients with endometriosis compared to healthy controls [319,320,321,322]. Most importantly, a large body of evidence suggests a significant reduction in intraovarian levels of several essential amino acids in patients with endometriosis. This is important because these amino acids are important for both oocyte competence and early embryonic development. These observations are consistent with recently published data showing that the ectopic development of endometrial tissue leads to extensive pelvic inflammation, which ultimately results in an impaired ovarian microenvironment [323]. Consequently, both oocyte competence and embryo developmental potential are impaired in endometriosis cases, leading to infertility and IVF failure.

In the same line, ovarian metabolism is significantly affected in PCOS. The metabolic footprint of PCOS pathophysiology and in particular PCOS-related insulin resistance in ovarian metabolism is characterized by increased levels of glucose, malate, oxaloacetate, and succinate and decreased levels of fumarate, lactate, and pyruvate in follicular fluid samples obtained from PCOS patients [318,324,325]. In addition, lipid metabolism and amino acid synthesis and secretion are significantly disturbed [318,324,325]. These findings strongly support the standing evidence suggesting that the first-line approach for effective management of PCOS-related infertility should be the regulation of glucose and lipid metabolism, which is significantly impaired in PCOS cases [326]. 

Regarding DOR, metabolomic studies analyzing follicular fluid samples from DOR patients indicate a significant dysregulation of pathways regulating lipid catabolism and amino acid metabolism. These findings provide a reasonable explanation for the observed increased oxidative stress in oocytes from DOR cases, as well as the compromised oocyte competence that subsequently leads to impaired embryo development [327,328,329,330]. In conclusion, analysis of the ovarian metabolism has provided important evidence to better understand and map the pathophysiological mechanisms leading to the development of common, albeit challenging to manage, causes of female infertility. These research efforts have already paved the way for the development of novel and sensitive metabolic biomarker panels to accurately assess the reproductive dynamic of individual cases.

Focusing on testicular metabolism and its impact on male infertility, major advances have been reported in the use of NMR/MS as a promising method for the assessment of various metabolites in semen, urine, and blood serum samples from infertile men [314]. Data suggest that the dysregulation of several metabolic pathways has a direct impact on spermatogenesis, endocrine regulation, and sex hormone synthesis and secretion, resulting in infertility [331,332]. More specifically, and in relation to abnormal spermatogenesis, pathways associated with redox balance and ROS production have been reported to be significantly impaired, namely, the urea cycle and the tricarboxylic acid cycle [333]. The impaired redox balance and extensive ROS production result in oxidative stress, representing the main cause of the observed increased prevalence of DNA fragmentation in several pathophysiological conditions leading to male infertility, such as varicocele [334]. Metabolism also plays a crucial role in the disruption of the endocrine milieu in male infertility. Several studies have demonstrated dysregulation of key metabolic pathways that regulate testosterone synthesis, secretion, and action, including the cytochrome P450 enzyme system, glucuronidation, and sulfation [335]. In addition, impaired functionality of other key enzymes involved in steroid hormone synthesis have also been reported in infertile men, including alterations in 17-alpha-hydroxylase [336]. 

Research into the identification of promising sensitive metabolic biomarkers for the assessment of testicular metabolism and male reproductive potential has yielded interesting results. Most of these studies have involved comprehensive metabolomic analysis of semen samples using high-throughput NMR/MS in infertile men with matched fertile populations as controls [337]. The data indicated that L-carnitine and acetyl L-carnitine hold significant potential as sensitive biomarkers for assessing testicular metabolism and functionality. These molecules are essential for sperm metabolism as they are used for energy production. In addition, both L-carnitine and acetyl L-carnitine play a crucial role in maintaining testicular redox balance and have antioxidant properties [338]. Moreover, several other metabolites have been characterized as potential biomarkers for assessing male fertility and sperm metabolic homeostasis, including citrate, fructose, lactate, pyruvate, and glucose [339]. Interestingly, seminal plasma metabolites have also been evaluated as potential biomarkers not only for assessing male fertility status, but also for predicting IVF outcome. Evidence suggests that seminal plasma levels of glutamate, lactate, pyruvate, fructose, and glucose may be used as promising biomarkers capable of predicting IVF success rates with adequate prognostic value [314]. As research in this field develops, the predictive value of several other metabolites is being investigated. For example, levels of antimycin A, glycine, ascorbate, and succinate have been reported to be positively associated with IVF success rates. In contrast, levels of nicotinate, threonate, and tyramine have been found to be negatively associated with IVF success [337]. 

In conclusion, metabolomic analysis of seminal plasma provides a non-invasive, accurate, and patient-friendly approach to assess testicular metabolism and male fertility status. Considering the impact of spermatozoa homeostasis on embryo developmental potential, seminal plasma biomarkers may also prove to be useful tools for predicting IVF outcomes. This is of particular importance, as testicular metabolomic analysis not only contributes to the development of personalized and precise diagnostic protocols, but also plays an important role in the design of an appropriate management strategy, allowing for interventions to optimize IVF efficiency.

Acknowledging the above mentioned, there is strong evidence that both ovarian and testicular metabolism are impaired in infertile individuals. The use of metabolomics has opened a new era in the identification of novel sensitive biomarkers for the diagnosis of infertility and the prediction of IVF outcomes. It has also been shown that there are metabolic biomarkers in follicular fluid and semen samples that can be used to assess embryo developmental capacity, since ovarian and testicular metabolic dysregulation affects gametes and subsequently the developmental dynamic of the resulting embryos. However, one of the most important “missing pieces of the puzzle” in optimizing IVF management is the selection of the embryo with the highest developmental potential and, most importantly, the identification of the optimal strategy for this selection in daily clinical practice [340].

Traditionally, decision-making on embryo selection has been based on the assessment of specific morphological criteria, including Gardner’s grading system for embryo selection at the blastocyst stage [341]. In the last decade, the use of sophisticated and novel culture systems in time-lapse incubators has opened a new era in embryo selection practice by introducing specific morphokinetic criteria. In practice, this means that the use of time-lapse incubators has enabled the analysis of both embryo morphology and developmental performance [342]. The additional use of artificial intelligence (AI) contributes significantly to the identification of the embryo with the highest implantation potential by reducing the subjectivity of human decision-making [343]. Despite these great advances, the optimal embryo selection strategy is still under investigation as IVF success rates remain relatively stable [344].

Over the past six years, it has been suggested that the solution to the puzzle of selecting the embryo with the highest developmental potential lies in the development of methods to accurately assess embryo physiology [345]. In this context, the study of embryo metabolic activity is a very promising approach as it can be performed non-invasively by analyzing spent culture media. The method of assessing the metabolic activity of cultured embryos to evaluate their developmental capacity was proposed as early as 1980, when glucose metabolism was suggested as a sensitive biomarker for assessing embryo viability [346]. In the following decades, several published studies showed inconclusive and contradictory results [347]. However, recent studies suggest that these discrepancies are mainly attributed to the research methodology being limited to the assessment of specific metabolic biomarkers. In contrast, the current use of metabolomics, including NMR/MS, has paved the way for the assessment of the whole embryo metabolic blueprint, providing interesting results [345].

Focusing on the metabolic activity of the embryo and its association with embryo viability and developmental competence, recently published data support that the glycolytic rate may be an important criterion for selecting the embryo with the highest implantation potential. The glycolytic rate expresses the amount of glucose consumption and lactate production and effectively indicates the relative rate of glycolysis. A high glycolytic rate is associated with poor embryo quality and competence [345,348,349,350]. A glycolytic rate of more than 100% indicates the use of endogenous metabolites such as glycogen and this is associated with poor implantation potential [345]. This is consistent with recently published metabolomic studies indicating that poor quality embryos with compromised developmental dynamic produce more cellular energy, presumably to correct possible cellular damage [351]. In addition, studies using metabolite set enrichment analysis and metabolomic pathway analysis indicate increased metabolic activity of biochemical pathways involved in mitochondrial and amino acid metabolism in poor quality embryos, supporting the above conclusions [351]. It has also been proposed that poor embryo quality is associated with increased levels of 2-hydroxyisovaleric acid and 2-hydroxyisocaproic acid in spent culture media. Both molecules are metabolites of valine, leucine, and isoleucine, defined as branched-chain amino acids, indicating an increased catabolic rate in poor-quality embryos [351]. Regarding free fatty acids, studies using MS profiling showed a statistically significant decrease in docosahexaenoic acid and an insignificant decrease in other essential free fatty acids in spent culture media where morphologically good-quality embryos were cultured [352]. These results suggest that high-competence embryos are biosynthetically active, in contrast to poor-quality embryos. This is also supported by data indicating decreased levels of phenylalanine, valine, proline, and tryptophan in spent culture media from embryos that were transferred, resulting in successful implantation [352]. 

Following the introduction of metabolomics in the era of developing novel and accurate methods for embryo selection, there is an open debate regarding the efficiency of these approaches, with some studies supporting their use and others reporting against. It is important to consider the possible reasons for this discrepancy. The first is the observed inconsistency between studies regarding the protocols used for metabolomic analysis. The second and most important reason is the presence of numerous confounding factors related to the embryo culture conditions. An increased heterogeneity between studies is observed with regard to the type of incubators, type of culture media, volume of culture media, oxygen tension, and several others. This is of paramount importance, as the use of metabolomic studies requires high levels of accuracy, precision, and reproducibility in all aspects. Therefore, in order to draw robust conclusions, the methodology for metabolomic analysis of spent culture media should first be standardized, and this is the main challenge that the scientific community should address in the future [345].

In conclusion, the use of metabolomics as a method to assess preimplantation embryo physiology is promising, although further studies are needed to provide conclusive and robust evidence before introducing these approaches into daily clinical practice. In the era of personalized and precision medicine, research efforts should be intensified towards the development of non-invasive tools for the combinatorial study of embryo morphokinetic performance, metabolome, and secretome using sophisticated artificial intelligence systems [353,354]. This can be achieved by using state-of-the-art “lab-on-a-chip” microfluidic systems that are currently under development. In the near future, these systems may become the “holy grail” of embryo culture incubators. Unlocking the secrets of embryo physiology may be the “missing piece” of the puzzle to optimizing IVF success rates and safety and ensuring the lifelong health of MAR individuals [345,355].

## 5. Discussion

Considering the current evidence, DNA methylation in male and female germ cells as well as in preimplantation embryos is likely to be determined by multiple factors. For the proper maintenance of different imprinted genes to be inherited normally and for proper gene regulation and expression, the physiological methylation process is crucial. In addition, maternal diet plays a pivotal role in the development and health of the newborn infant [356]. In the Maternal Nutrition and Offspring’s Epigenome (MANOE) study, it was suggested that maternal dietary intake and supplementation of methyl donors may have an impact on the infant’s DNA methylation of genes involved in metabolism, growth, appetite regulation, and maintenance of DNA methylation reactions [356]. Alterations in one-carbon metabolism, and subsequently to DNA methylation, appear to have long-lasting implications for the newborn’s health as well as the health of the mother.

Given the clinical importance of the alteration of DNA methylation in gametes and preimplantation embryos, several studies investigate the relationship between methyl donor availability and MAR procedures. It is widely accepted that infertile patients are more likely to present with DNA methylation errors attributed to infertility [122]. However, only a limited number of studies have compared the availability and supplementation of methyl donors between infertile and fertile populations. The effect of methyl donors in embryo development remains an important question, as they could be either supplemented to diet or employed as add-ons to IVF culture media. 

The composition of culture media may require reevaluation in light of the most recent knowledge on embryo physiology as MAR-associated disruption of the folate and methionine cycle can change DNA methylation to a critical point for the oocyte’s maturation or for the embryo’s development [357,358]. Medium enrichment with methyl donors to maintain the proper DNA methylation pattern in gametes and preimplantation embryos could be considered. Zinc could also be considered as an add-on for culture media, especially regarding cryoprotectants for sperm, as it seems to have an ameliorative effect over oxidative agents [249]. According to the studies presented herein, addition of zinc preserved sperm chromatin integrity in humans. According to animal studies, its employment in oocyte maturation as well as sperm preparation medium improved IVF outcomes significantly [232]. However, this could solely be employed as an indication for further studies in humans. The role of folate supplementation remains to be investigated with studies presenting controversial results. However, its critical role seems to be validated by the fact that *MTHFR* mutations lead to lower number of oocytes retrieved and embryos available for transfer. Nonetheless, further studies are required to delineate whether the levels of folate could be associated with IVF outcome. 

The regulation of 1-C metabolic pathways is mostly controlled by various vitamins, and the most crucial appears to be vitamin B12 [273]. It has been suggested that the availability of folate and vitamin B12 in infertile patients may be associated with higher chances of live birth [359]. In relation to studies on choline, what becomes clear is that it is an essential methyl donor that protects against neural and metabolic defects during embryo and fetal development [284]. In addition and considering 1-C metabolism, betaine and choline play an important role in the methionine cycle, especially in mitochondria, by increasing methionine and SAM availability [296]. Despite the fact that only one relevant study was identified concerning humans, studies based on animal models report that betaine-supplemented culture media have protective effects over ROS production that clinically can be useful in cases of FASD [301]. Homocysteine, being the last molecule in the methionine cycle of the 1-C metabolism, can serve as a biomarker regarding embryo developmental potential. While a negative association with Hcy levels and reproductive outcomes has been observed, further studies are required to evaluate its predictive capabilities.

One would expect that the answer would be the availability of these substrates; however, balance is key. Indicatively, supplementation of zinc and folate to the male partner does not seem to enhance pregnancy outcomes [268]. As has been observed in both males and females, these nutrients present with optimal effect at specific levels [234]. Perhaps to avoid any possible adverse or detrimental effects, the nutrients could be employed principally for culture media enhancement and not be prescribed to infertile patients. It may be of importance for the scientific community to exercise extra caution when considering prescription to infertile patients, while evaluation of baseline levels prior to supplement administration may be required. A possible role for Hcy could be that of a biomarker to evaluate the possibility of achieving a pregnancy and possibly through further research to be employed in the formulation of the culture media, aiming to achieve optimal embryo development. 

Data revealing connections between various methyl donors, gametes, embryo DNA methylation, and MAR procedures are collectively analyzed in this review, which may appear to lead to the conclusion that MAR plays a substantial role contributing to DNA methylation disruption. Understanding the mechanisms underlying DNA methylation is of critical importance in order to apply treatment modalities for disorders of the male and female genital system caused by abnormal DNA methylation [360]. To shed light on the mechanisms underlying these consequences, carefully designed clinical studies in conjunction with appropriate animal model studies are required. Studying the interactions and ideal levels of the various methyl donors on both gametes and embryos may result in the development of more beneficial culture media employed in IVF, as well as establishment of potential biomarkers for their quality and, subsequently, IVF outcomes. However, to confirm this, further studies are required to evaluate the possible role of these nutrients in culture media, conditions, and possibly to assist in developing individual protocols according to the need of each couple abiding by the principles of personalized medicine.

Considering the data and critical analysis presented herein, it should be noted that this study presents with limitations and reasons for caution. This study is a narrative review of the literature, and this is associated with possible selection bias with regard to the criteria employed during the process of study selection. A comprehensive screening and data collection process was employed to minimize the respective bias associated with the narrative nature of this review. The conduction of a systematic review and/or meta-analysis in this study’s topic was considered immature, since limited data with regard to the clinical utility of 1-C metabolism in MAR exist. Moreover, and as previously indicated, the data presented herein mainly originate from studies characterized by small and heterogenous populations. Heterogeneity was also observed with regard to interventions performed, since different methodologies were observed among the studies discussed herein. However, this narrative review indicates that it is of importance to intensify the research towards unveiling the possible implications of 1-C metabolism in MAR, since robust evidence indicates that 1-C metabolic dysfunction is associated with infertility, MAR efficiency, and, most importantly, with the lifelong health of MAR infants.

## 6. Conclusions

It is certain that the metabolic profile of the embryo defines its developmental potential [192]. Metaboloepigenetics appears to be a promising field that could bring together research focused on optimization with regard to the creation and culture of embryos in IVF labs, with research further focused on identifying the optimal selection tool to indicate the embryo with the highest implantation potential. Interestingly, the old and revisited question remains: “Can the metabolic profile of an embryo be employed as a tool to predict clinical outcomes?” Hitherto, current—albeit limited—evidence seems to fail to suggest an association between employing the metabolomic profile as a prognostic tool and clinical outcomes [361]. With regard to the critical analysis performed herein on the value of methyl donors, it appears that assessing and optimizing the levels of methyl donors and of molecules intertwined with the 1-C metabolism pathway, an improvement in culture media and conditions may be achieved, indicating that there is room and incentive to investigate this aspect. When considering investigating certain fields of research in MAR, the driver should be dynamic, acknowledging the importance of not only improving clinical pregnancy and live birth rates, but also the significance of achieving optimal perinatal, neonatal, and pediatric outcomes, ensuring the lifelong health of MAR infants.

## Figures and Tables

**Table 1:** Summary of studies reporting on clinical implications of zinc in medically assisted reproduction.

Study	Intervention/Observation	Outcome Measure	Results
**Komiya et al., 2023** [210]	Zinc serum levels in males	Total motile sperm count	NS
**Dadgar et al., 2022** [211]	Zinc supplementation in males	Semen parameters and Sperm DNA fragmentation	Increased normal spermatozoa morphology and decreased DNA fragmentation index following zinc supplementation
**Chabchoub et al., 2021** [212]	Zinc serum levels in males	Zinc levels in fertile vs. infertile men	Higher in fertile men (cut-off value: 111.8 µg/dL, AUC: 0.928)
**Wang et al., 2021** [213]	Zinc serum levels in females	Adverse in vitro fertilization outcomes (failure to achieve clinical pregnancy)	Lower zinc levels were associated with lower clinical pregnancy rates
**Schisterman et al., 2020** [214]	Zinc and folic acid supplementation in males	Multiple reproductive outcomes	NS
**Tulic et al., 2019** [215]	Zinc serum levels in females	Deliveries and miscarriages	NS
**Wdowiak et al., 2018** [216]	Zinc follicular fluid levels	Clinical pregnancy	NS
**Berkovitz et al., 2018** [217]	Addition of zinc in cryoprotection media	Semen parameters	Statistical analysis was not performed
**Ingle et al., 2017** [218]	Follicular fluid and urine zinc levels	Multiple reproductive outcomes	Negative correlation between follicular fluid zinc levels and fertilization rate; Positive correlation between urine zinc levels and number of available embryos
**Isaac et al., 2017** [219]	Addition of zinc oxide nanoparticles in cryoprotection media	Post-thaw semen parameters and chromatin integrity	Lower chromatin damage following addition of zinc oxide nanoparticles in cryoprotectant media
**Giacone et al., 2017** [220]	Addition of zinc, D-aspartic acid, and coenzyme Q10 in sperm culture media	Progressive motility following 3 h of incubation and swim-up	Addition of zinc, D-aspartic acid, and coenzyme Q10 to sperm culture media following 3 h incubation resulted in increased progressive motility in asthenozoospermic men; following swim-up increased progressive motility in all samples
**Nematollahi-Mahani et al., 2014** [221]	Zinc sulfate supplementation and zinc sulfate plus folic acid supplementation following varicocelectomy	Sperm parameters	Zinc sulfate supplementation increased sperm normal morphology; zinc sulfate plus folic acid supplementation increases sperm concentration, progressive motility, and normal morphology
**Singh et al., 2013** [222]	Follicular fluid zinc levels	Endometriosis vs. tubal factor infertility; pregnancy within groups	Lower level of zinc in endometriosis group; higher levels of zinc in women with endometriosis who achieved pregnancy
**Kotdawala et al., 2012** [223]	Addition of zinc in cryoprotection media	Post-thaw sperm parameters and chromatin integrity	Increased progressive motility and chromatin integrity following addition of zinc
**Atig et al., 2012** [224]	Seminal plasma zinc levels	Sperm parameters	Positive correlation with sperm concentration and motility; negative correlation with normal morphology
**Dickerson et al., 2011** [225]	Hair and serum zinc levels in females	Follicle number and oocyte yield	Positive correlation of hair zinc levels with oocyte yield; no correlation between hair and serum zinc levels
**Colagar et al., 2009** [226]	Seminal plasma zinc levels	Zinc levels in fertile vs. infertile men	Fertile men presented with higher zinc levels compared to those infertile
**Omu et al., 2008** [227]	Zinc supplementation alone or in combination with other vitamins in males	Semen parameters and DNA fragmentation index	Zinc supplementation alone or in combination with other vitamins increased sperm parameters and decreased DNA fragmentation index
**Ebisch et al., 2006** [228]	Zinc supplementation in males	Semen parameters and reproductive hormone levels	Zinc supplementation increased sperm concentration
**Benoff et al., 1999** [229]	Seminal plasma zinc levels	Semen parameters; fertilization and clinical pregnancy rates	NS
**Tikkiwal et al., 1987** [230]	Supplementation of zinc in males	Semen parameters	Increase in sperm count, progressive motility and normal morphology

NS: non-statistically significant difference.

**Table 2:** Summary of studies reporting on clinical implications of folate in medically assisted reproduction.

Study	Intervention/Observation	Outcome Measure	Results
**De Cosmi et al., 2023** [257]	Serum folate levels in females	Multiple IVF outcomes	NS
**Polzikov et al., 2022** [256]	Serum folate levels in females (comparison between highest and lowest quantiles of folate concentration)	Number of oocytes retrieved, clinical pregnancy, and live birth	Women in the highest quantile presented with decreased oocyte yield and decreased odds for clinical pregnancy and live birth
**Tabatabaie et al., 2022** [258]	Supplementation of folate and folate with myoinositol in polycystic ovarian syndrome cases	Number of oocytes retrieved, MII rate, fertilization rate, and embryo quality	Only women receiving myoinositol and folate presented with increased oocyte yield, MII and fertilization rate, and embryo quality compared to both other groups
**D’Argent et al., 2021** [259]	Supplementation of folate in males	Sperm parameters, sperm DNA fragmentation, positive hCG, and clinical pregnancy	Decrease in sperm DNA fragmentation, increase in positive hCG rate. NS in sperm parameters and clinical pregnancy rate
**Mohammadi et al., 2021** [260]	Supplementation of folate and folate with myoinositol in poor ovarian response cases	Number of oocytes retrieved, MII rate, fertilization rate, embryo quality, clinical pregnancy, and live-birth	NS
**So et al., 2020** [261]	Supplementation of folate with L-arginine in women	Positive human chorionic gonadotropin test and clinical pregnancy	NS (statistical significance was observed in subgroup analysis, albeit with significantly wide confidence intervals)
**Nazari et al., 2020** [262]	Supplementation of folate with myoinositol in women	Number of retrieved oocytes, embryo quality, fertilization, implantation, and ongoing pregnancy rates	Increased fertilization, good-quality embryo, implantation, and ongoing pregnancy rates
**Regidor et al., 2018** [263]	Supplementation of folate with myoinositol in women	Number of retrieved oocytes, embryo quality, and fertilization rates	Improved fertilization and embryo quality rates
**Nouri et al., 2017** [264]	Supplementation of folate among other nutrients	Embryo quality on day 3 and clinical pregnancy rate	Improved embryo quality on day 3
**Murto et al., 2014** [265]	Supplementation of folate in women	Clinical pregnancy	NS

NS: non-statistically significant difference.

**Table 3:** Summary of studies reporting on clinical implications of homocysteine in medically assisted reproduction.

Study	Intervention/Observation	Outcome Measure	Results
**Manzur et al., 2023** [309]	Homocysteine and B12 serum levels in females	Number of embryos available for transfer	Homocysteine levels were negatively associated with number of embryos available for transfer
**Wang et al., 2022** [310]	Homocysteine serum levels in poor ovarian response cases	Embryo quality	Negative association between homocysteine levels and embryo quality
**Chen et al., 2021** [311]	Homocysteine serum levels in females	Clinical pregnancy	Negative association between homocysteine levels and clinical pregnancy rates
**Razi et al., 2021** [307]	Homocysteine follicular fluid levels	Oocyte maturity, embryo quality, and clinical pregnancy	Negative association between homocysteine levels and oocyte maturity, and embryo quality and clinical pregnancy rates
**Liu et al., 2020** [312]	Homocysteine serum levels in females	Multiple reproductive outcomes	Only negative association with number of oocytes retrieved
**Berker et al., 2009** [302]	Homocysteine follicular fluid levels	Multiple reproductive outcomes	Negative association with fertilization rate and embryo quality
